# The Catholicity of Medicine

**Published:** 1916-08-05

**Authors:** 


### The Hospital

The Workers' Newspaper of Administrative Medicine and Institutional Life, Administration, National Insurance and Health.

No. 1574, Vol. LX. SATURDAY, AUGUST 5, 1916. PRICE ONE PENNY.

### THE CATHOLICITY OF MEDICINE.

The mental atmosphere which is provided by a man's occupation is primarily a matter of importance to himself. It may cramp and confine him on the one hand, or may stimulate him to growth and expansion on the other. Probably the very strongest and most resolute utilise their environment whatever be its character, but the majority feel the pressure of their surroundings and even yield to its sway. Hence in habits of thought and modes of expression men often reveal their callings, and the observant person frames on such incidents a fairly confident conclusion. The truth of all this is of some importance to the individual in the selection of a profession, though in such selection it is by no means always a weighty factor. It is of importance, also, to the community, as the atmosphere of a man's life influences—and perhaps determines—his relations to others and his outlook on large questions which are a matter of common concern. The fashion of his daily thought imprints itself on outside and occasional issues, and what is done, as the phrase runs, on "the spur of the moment" is an outcome of the unconscious preparation practised daily over many years. The swift summons brings to the surface what has been made ready by sustained habit and needs but the event to reveal it. In this sense, therefore, other considerations apart, the mental atmosphere of medical study and practice is a subject of widespread interest. If primarily it concerns those who follow the calling, it none the less reaches to the intelligent outsider.

To some it may seem a bold thing to claim that medicine offers to its disciples a liberal, generous, and catholic mental life. There are those who would confidently deny the proposition, and would, indeed, affirm that the truth lies at or near the opposite pole. Not a few people quite honestly believe that the medical practitioner is in bonds to custom, to etiquette, and to prejudice, if not to rule and authority. His procedure, and more or less his habits of thought, are, it is held, fashioned by a rigid code which moves his actions and colours his judgment. It is tradition, rather than the freedom of the individual, which is the governing factor in the world of medicine, and woe be to him who cultivates and asserts a personal view. This, or something like this, is a widespread popular doctrine, though perhaps it is less clearly defined and less loosely held than the words here written would suggest, and it is fair to add that in practice such a view is applied to the profession as a whole rather than to any one of its individual representatives. The memory of much kindliness and helpfulness redeems the individual practitioner from any severity of criticism by those whom he has served, and he is credited with being better than his creed. None the less, his creed and his class are often regarded as stamped by narrowness and exclusiveness.

There is for those who will hear it a full answer to all these reproaches. In the first place there is an *a priori* improbability in such charges, seeing that medical education and training are conducted in a scientific setting, where there exists without question a stimulus to freedom of judgment and to unrestrained debate. Indeed, there are those who hold that this freedom is excessive, and that under its influence all that boasts the sanction of authority and tradition melts into thin air. In any event there remains the truth that in his early and flexible days the student of medicine lives among influences which teach him the value of truth from whatever source this comes, and which accustom him to respect theory and practice in so far, and only in so far, as these can justify themselves by the test of evidence and demonstration. Is it likely that out of such an atmosphere will grow a blind and obstinate attachment to formulas and a willingness to submit the mind to mere traditional and official utterances? Rather it is to be expected that, while impatient of pretence and sham and unreality, the practitioner will insist upon his right to freedom of thought, and will naturally and habitually reject any attempt to bind him to customs and practices which are innocent of sound justification. This, we repeat, is the probability inherent in the circumstances. In our judgment it is confirmed by experience, but even those who challenge such a conclusion must admit that the result which they describe is an unexpected and a surprising one.

Perhaps one of the principal causes of such adverse conclusion is the custom of the profession to regard with some suspicion suggestions—and especially it may be therapeutic suggestions—taking origin from extra-professional sources. There is no need to deny some degree of impatience in this respect. Medical practitioners, like other human beings, feel the bias of their occupations and the expert, whatever be his field, is inclined to look askance at amateur proposals. As a result, it may be that in occasional instances he misses a suggestion having some degree of value. But against such exceptional loss is to be set the consideration that the patient and impartial study of all proposals owning a non-professional origin would mean much weariness of spirit and a result quite incommensurate with the time which the process involved. After all, it must be remembered that life is short and that work has to be done. What is best in the long run and on the whole is the sound motive for intelligent persons who recognise that mistakes will occur whatever the method and that for each worker the night surely cometh. Hence there is need for impatience with those whose intervention will cause delay, and in all probability fruitless delay, in the accomplishment of tasks which urgently await achievement. It is not from mere obstinacy and prejudice that the medical profession seems often to turn a deaf ear to untrained advisers. The procedure is based upon experience and on the balance of probability. Such action is a method of protection dictated by common sense, and it cannot be quoted against the claim that a readiness to welcome new truths is an inevitable part of the catholic discipline of medicine.

